# Oral lesions associated with nevirapine-related Stevens Johnson syndrome: A report of four cases

**DOI:** 10.4103/0973-029X.80024

**Published:** 2011

**Authors:** S Balasundaram, K Ranganathan, K Umadevi, R Gunaseelan, N Kumaraswamy, Sunithi Solomon, Bella Devaleenol, Pradeep Ambrose

**Affiliations:** *Departments of Oral Pathology, Chennai Dental Research Foundation, Chennai, Tamil Nadu, India*; 1 *Ragas Dental College and Hospitals, Chennai, India*; 2 *Chennai Dental Research Foundation, Chennai, India*; 3 *YRGCare, Chennai, India*

**Keywords:** HIV, Nevirapine, oral lesions, Steven Johnson syndrome, ulcers

## Abstract

Nevirapine is a non-nucleoside reverse transcriptase inhibitor, widely used in combination with other antiretroviral agents for treatment of HIV infection. Steven Johnson syndrome (SJS) is the major toxicity of nevirapine. We describe here four cases of SJS in HIV seropositive patients following nevirapine therapy. In all four cases cutaneous hypersensitivity reaction was seen with extreme oral lesions, three patients presented clinically with elevated liver enzymes and hepatitis, and two patients had ocular involvement.

## INTRODUCTION

Stevens Johnson syndrome (SJS) is a severe hypersensitive reaction that can be precipitated by infection, vaccination, systemic diseases, physical agents, foods and drugs.[1,2] The drugs that cause SJS commonly are antibacterials (sulfonamides), anticonvulsants (phenytoin, phenobarbital, carbamazepine), non-steroidal anti-inflammatory drugs (oxicam derivatives) and oxide inhibitors (allopurinol).[3,4] SJS may present as a nonspecific febrile illness (malaise, headache, cough, rhinorrhea) with polymorphic lesions of skin and mucous membrane characterized by acute blisters and erosions.[2] One of the undesirable side-effects of highly active anti-retroviral therapy (HAART) in HIV management is SJS. In this article, we report oral lesions associated with nevirapine (NVP)-related SJS in four HIV seropositive patients seen at a tertiary HIV care centre in Chennai, India.

## CASE REPORTS

### Case 1

**Figure 1:**
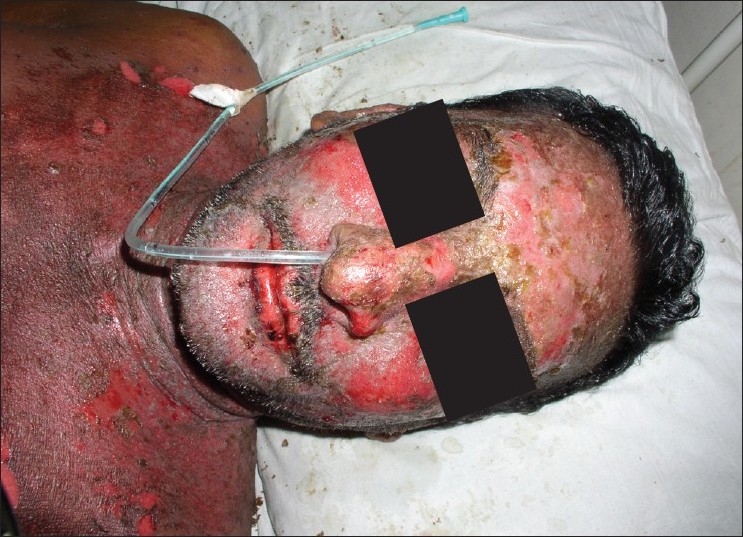
Pre treatment – ulcers, hemorrhagic erosions in the trunk, face (Case 1)

**Figure 2:**
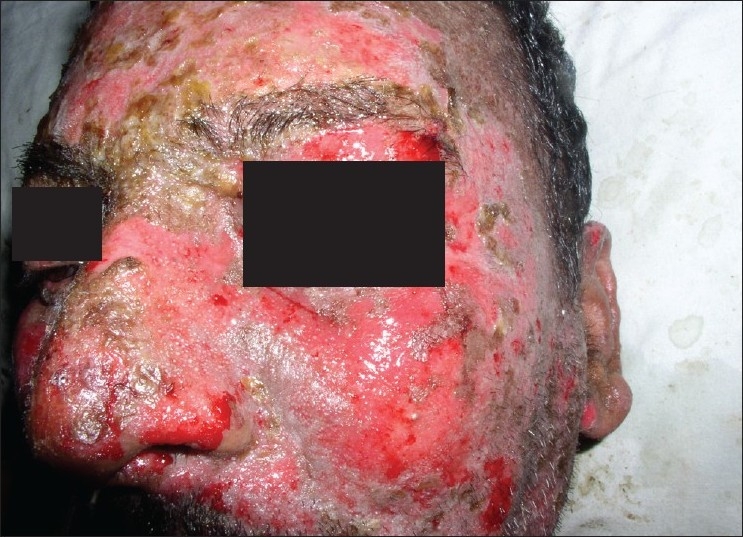
Pre treatment – ulcers in theupper and lower lip (Case 1)

A 50-year-old male, presented with complaints of fever and extensive rashes on the skin of the face and the neck, ulcerations and erythema of the conjunctiva and the oral cavity and difficulty in swallowing, of ten days’ duration. The past medical history of the patient revealed that he had been diagnosed with HIV infection (HIV-1) eight months back. He was initiated on HAART therapy (zidovudine 300 mg + lamivudine 150 mg + NVP 200 mg) a month back when the viral load was 1, 09,000 copies/ml. The patient was also on anti-tuberculosis treatment (ATT) (rifampacin 150 mg, isoniazid 300 mg) and vitamin supplements for the past three months. The patient was well-oriented and on examination, had hyperpyrexia, generalized, maculopapular and bullous eruptions on the neck, face and the trunk [Figures 1 and 2]. Intraorally, the patient was completely edentulous and had multiple oral ulcers of the buccal mucosa, soft palate and buccal vestibule. The ulcers were hemorrhagic and tender on palpation. Hemorrhagic erosions were also seen on both the upper and lower lips [Figures 1 and 2]. Ophthalmic examination showed acute conjunctivitis and subconjunctival hemorrhages. There was no history of previous hypersensitivity reaction to drugs. The pruritis involving the oral and the ocular region was followed by vesicles and ulcerations four days after the initiation of HAART therapy.

The laboratory investigations at the time of admission to our referral centre showed a CD4 count of 470 cells/microliter (normal range 200-1347 cells/microliter), total white blood cell (WBC) count was 4860-cells/cu mm (normal range 4000-11000 cells/cu mm), hemoglobin 11.9 g/dl (normal range 12-17 g/dl) and erythrocyte sedimentation rate (ESR) 36 mm/h (normal range 0-14mm/h). The platelet count was 208 × 10^9^/L (normal range 137-367 × 10^9^/L) and random blood glucose was 186 mg/dl (normal range 80-120 mg/dl). Urine analysis and serum chemistry were within normal limits. A chest radiograph of the patient did not show any active tuberculous lesion.

**Figure 3:**
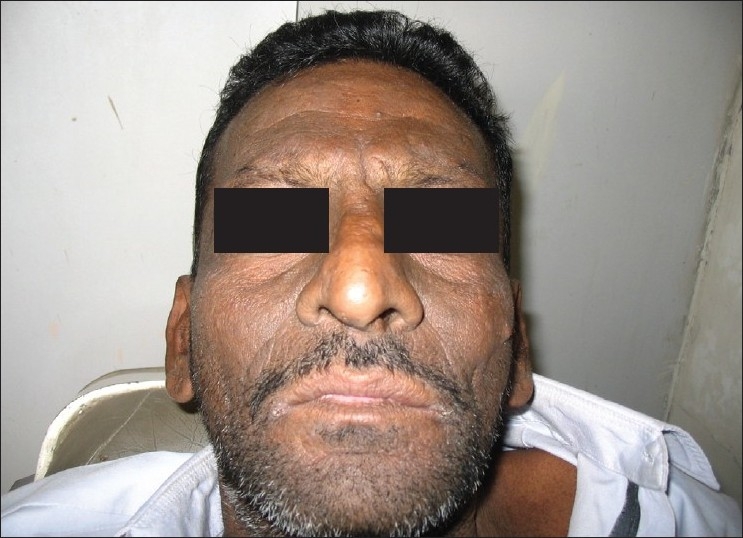
Post treatment – Skin lesions resolved (Case 1)

**Figure 4:**
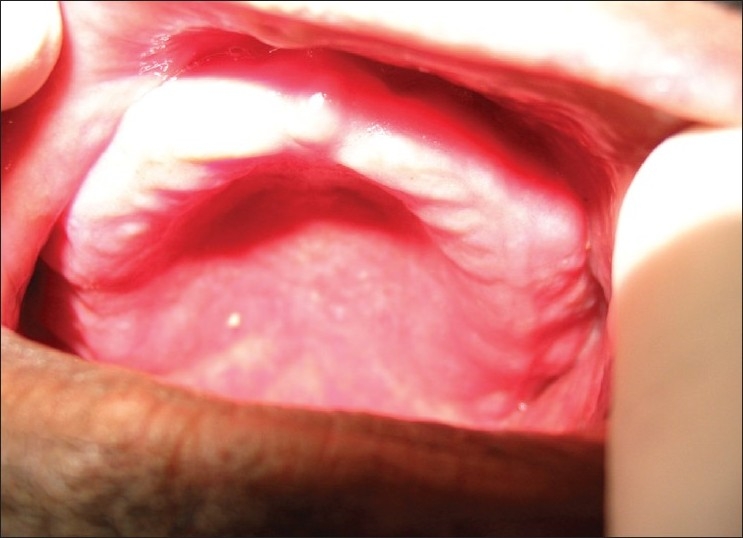
Post treatment – Oral mucosal lesions resolved (Case 1)

On the basis of the history and clinical presentation a diagnosis of SJS was made. As the first line of treatment HAART was discontinued and the patient was administered intravenous 5% dextrose and 2 ml dexamethasone (4 mg/ml). Supportive therapy with oral topical anesthetic gel (lignocaine 2%) for the oral ulcers was prescribed. The ocular lesions were managed with dexamethasone (0.1%) eye drops and crusting dry lesions were managed with liquid paraffin. The skin and the oral lesions healed over a period of two weeks. The patient was subsequently followed up and after 12 weeks the oral mucosal lesions resolved completely [Figures 3 and 4].

### Case 2

**Figure 5:**
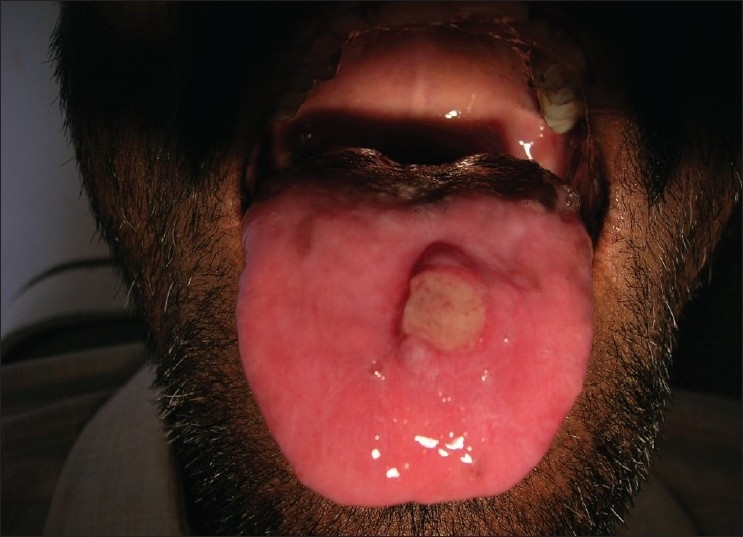
Pre treatment – ulcers in the tongue (Case 2)

A 39-year-old HIV seropositive patient reported with breathlessness, fatigue, fever, and abdominal pain of five days’ duration. On general examination the patient was well-oriented and conscious. The past medical history revealed that the patient was diagnosed as HIV seropositive (HIV 1 and 2) two months back. He was being treated for pulmonary tuberculosis (rifampacin 150 mg, isoniazid 300 mg) for the past two months. He was also on topical antibiotics for the past one month for the management of otitis media. Two weeks prior to reporting to us, he was started on HAART (lamivudine 150 mg, stavudine 30 mg and NVP 200 mg) and five days following this he developed mucocutaneous rash. There was no previous history of allergic reaction to drugs. Physical examination revealed generalized skin rashes on the neck and the trunk. Abdominal pain was thoroughly evaluated clinically by the physician. On intraoral examination, there was inflammation of the buccal mucosa and the palate. The labial mucosa was erythematous and tender. Oral Candidiasis (OC) was seen on the palate and the tongue [Figure 5].

The routine laboratory investigations showed an ESR of 49 mm/h, hemoglobin-9.2 g/dl and the differential count showed a raised eosinophil count to 6%. A clinical diagnosis of SJS and NVP-induced hepatitis was made based on the history and clinical presentation. HAART was discontinued and 2 ml dexamethasone (4 mg/ml) was given to manage mucocutaneous rashes and intravenous fluids were also administered. OC was treated with topical clotrimazole. Vitamin supplements were also prescribed.

The patient was subsequently followed and discharged after one week, as his condition was stable. He was advised to continue ATT and vitamin supplements. Two months later ART was initiated with oral lamivudine 150 mg and efavirenz 600 mg, without rechallenge of NVP. A follow-up of the patient for six months confirmed that he was responding to ART and had not developed any drug reaction.

### Case 3

**Figure 6:**
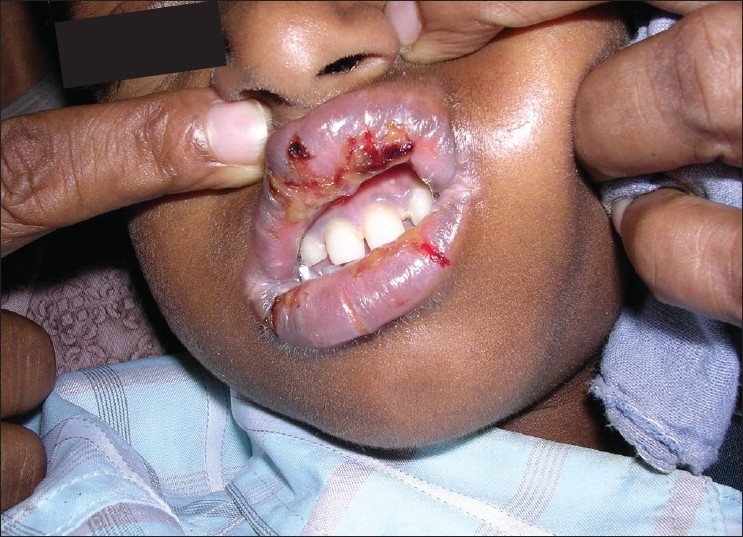
Pre treatment – ulcers in the upper lip (Case 3)

**Figure 7:**
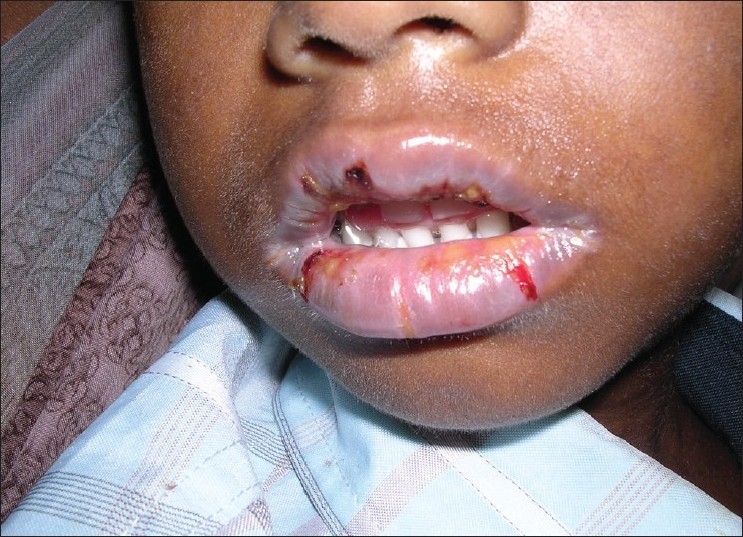
Pre treatment – ulcers in the lower lip (Case 3)

A four-year-old boy was admitted with complaints of fever, abdominal swelling and difficulty in passing urine for the past ten days. On general examination, he was well-oriented and conscious. The medical history revealed that the patient was diagnosed with HIV infection two years back and was on ATT for the past two years. One month back the patient was started on ART (syp Lamivudine 2.5 ml and syp NVP 2.5 ml). Along with ART, the patient was advised to continue ATT (rifampacin 150 mg, isoniazid 300 mg) and vitamin supplements. The patient reported to us three weeks after the initiation of ART with complaints of ulcers on the lip, pedal edema and cutaneous rashes all over the body [Figures 6 and 7]. On intra-oral examination extensive ulcerations of the lip and the tongue were seen. Ophthalmic examination revealed bilateral conjunctivitis. The CD4 count at the time of admission was 215 cells/microliter and further declined to 36 cells/microliter five days later. The patient was started on intravenous 5% dextrose, Syp Cetrezine hydrochloride 2.5 ml thrice daily for five days.

**Figure 8:**
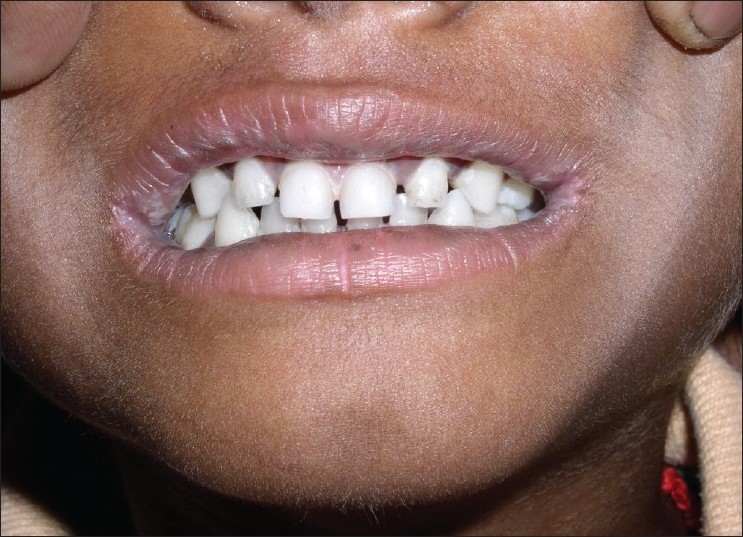
Post treatment – lip lesions resolved (Case 3)

**Figure 9:**
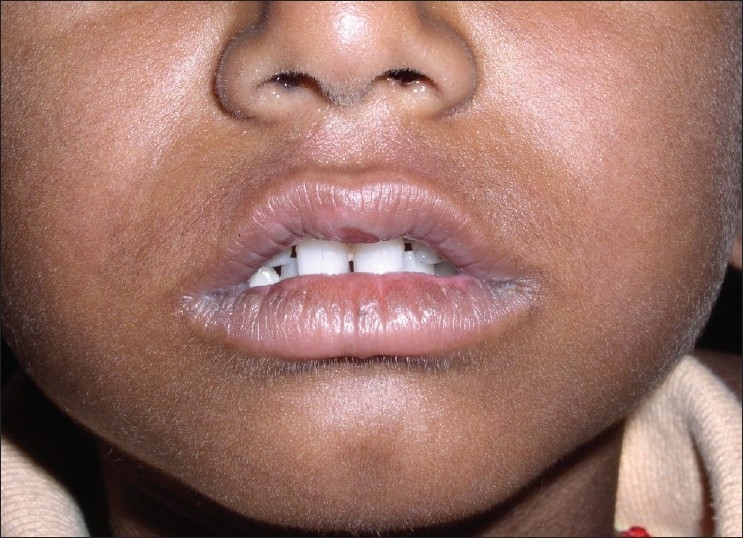
Post treatment – lip lesions resolved (Case 3)

Laboratory investigations showed, hemoglobin 8.2 g/dl, ESR 17 mm/h, increased serum bilirubin-1.7 mg/dl (normal range 0.3-1.2mg/dl), and gamma glutamyl transpeptidase 120 IU/L (normal range 11-50 IU/L). The albumin level declined to 2.7 g/dl (normal range 4.2-5.2 g/dl). The abdominal pain was evaluated by a gastroenterologist. Based on the clinical and laboratory findings a diagnosis of SJS and hepatitis was made. There was an increased serum level of bilirubin and decreased albumin level. The patient was started on ursodeoxycholic acid 150 mg twice daily, alkaline citrate 1.4 g/5 ml suspension, to manage the hepatitis. The skin and the oral lesions were managed symptomatically and high-protein supplements were included in the diet. As his condition was stable and since the patient did not develop any further hypersensitive reaction he was subsequently discharged after one week. HAART (laminivir, stavudine and efavirenz) was initiated again and continued without rechallenge of NVP. The patient was followed up regularly for six months and the response to the treatment [Figures 8 and 9] was uneventful and satisfactory.

### Case 4

**Figure 10:**
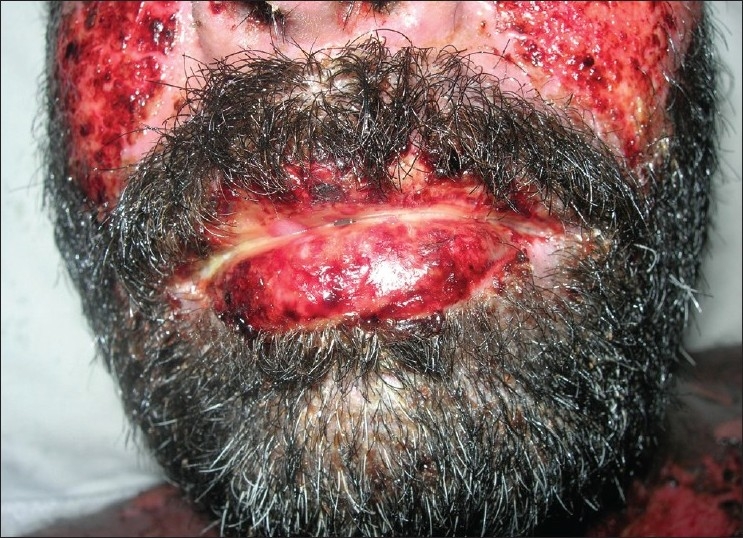
Pre treatment – ulcers and hemorrahagic lesions on the face (Case 4)

**Figure 11:**
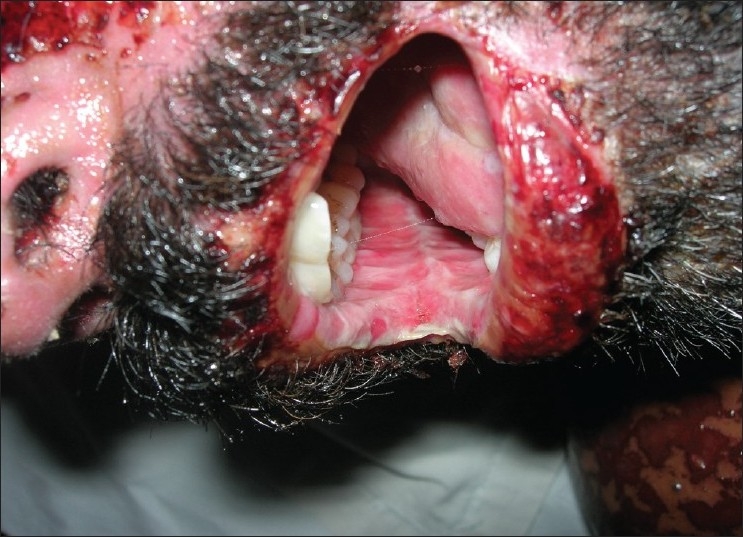
Pre treatment – ulcers on buccal mucosa (Case 4)

**Figure 12:**
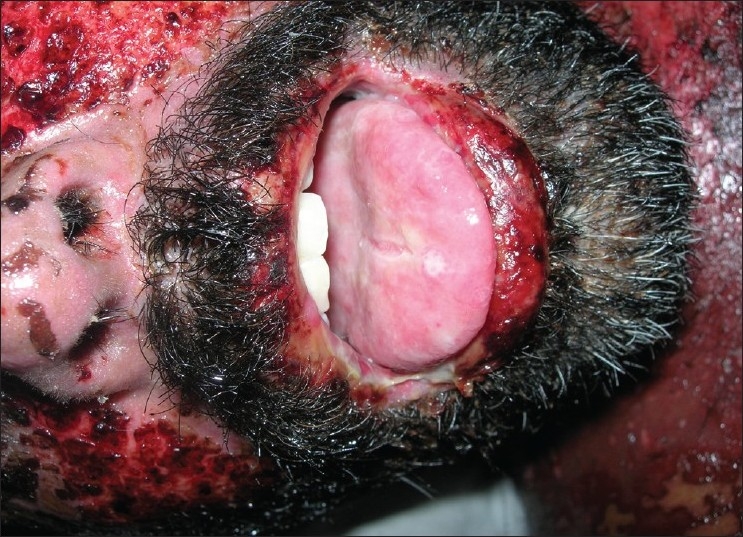
Pre treatment – ulcers on tongue (Case 4)

A 39-year-old man presented with complaints of fever, extensive rashes of the face and the neck. Ulcerations of the oral cavity and of the ocular regions were of one-week duration [Figures 10, 11 and 12]. The patient was diagnosed to be HIV seropositive three months back. He gave a history of being treated for rheumatic heart disease and mitral stenosis with associated pulmonary hypertension, for the past 10 years with digoxin 0.25 mg and verapamil 120 mg twice daily. The patient was a known diabetic and his hyperglycemic status was managed by glipizide (sulphonylurea) 2.5 mg daily. Fifteen days back he was initiated on HAART (lamivudine 150 mg, stavudine 30 mg and NVP 200 mg). He developed hyperpyrexia followed by pruritis and oral ulcerations four to five days after the initiation of HAART. He did not have any previous history of hypersensitivity to drugs.

On general examination, the patient was well-oriented and conscious. On admission the patient had hyperpyrexia, generalized, maculopapular cutaneous eruptions of the neck, face and the trunk. Intra-oral examination revealed multiple oral ulcers that were tender and hemorrhagic erosions were present on both the upper and the lower lips. Ophthalmic examination revealed conjunctivitis and diffuse erythema on the upper eyelids.

Laboratory investigation at the time of presentation showed a CD4 count level of 728 cells/microliter, WBC total count 15.3 ×10^9^/L, hemoglobin 14.2 g/dl, ESR- 95 mm/h and platelet count 401×10^9^/L. The random blood glucose was 186 mg/dl. The liver function test showed elevated gamma glutamyl transpeptidase of 295 IU/L (normal range 11-50 IU/L) on admission, and this reduced to 158 IU/L after he was off NVP for one week. Urine analysis was apparently normal.

The generalized maculopapular cutaneous eruptions and the liver function tests were suggestive of SJS and hepatitis. HAART was discontinued and the patient was subsequently managed symptomatically by administering intravenous 5% dextrose, inj dexamethasone 2 ml (4 mg/ml), inj ranitidine 2 ml (25 mg/ml), inj metronidazole 100 ml (0.5%w/v), Cetrezine hydrochloride (10 mg twice daily), calcium channel blocker-verapamil (120 mg twice daily), cardiac glycoside-digoxin (0.25 mg) and antacid gel were administered by oral route. The oral lesions were treated with topical lignocaine gel and chlorhexidine mouth rinse. Ocular lesions were treated by the administration of dexamethasone (0.1%) eye drops. The patient responded to therapy and after four weeks the skin and the oral lesions had resolved.

## DISCUSSION

Oral lesions are a feature of HIV infection and our earlier reports from India have documented ulcers in patients who were not on antiretroviral therapy.[5] Generic HAART is now available in India and with the advent of HAART there is a change in the profile of oral lesions.[6] In our cohort of 3329 patients 18% (*n*=614) patients were on HAART and here we report our experience of SJS in four patients who were on HAART.[5,7] An increasing number of medications and regimens are used in the management of HIV infection. Oral lesions in HIV infection due to drug reactions present a life-threatening barrier to the effective treatment of HIV infection and AIDS. Many studies have confirmed a higher incidence of adverse drug reactions among patients with HIV/AIDS than in the general population.[8,9] The increasing incidence of multiple drug reactions is also well documented in HIV-infected patients.[6,10] Identification of a single antiretroviral drug as the cause of a drug eruption in a patient infected with HIV-1 is often difficult because antiretroviral drugs are rarely used as monotherapy.[11] A typical antiretroviral combination consists of two drugs, termed antiretroviral therapy (ART) or a three-drug combination known as HAART. It comprises one nucleoside reverse transcriptase inhibitor (NRTI) + two non-nucleoside reverse transcriptase inhibitors (NNRTIs) or a protease inhibitor (PI). Additionally, the patients may also be on other medications to manage opportunistic infections.

SJS and toxic epidermal necrosis (TEN) are severe cutaneous disorders characterized by acute skin blisters and mucous membrane erosions. In TEN, necrosis of the epidermis and other epithelia are seen. The distinguishing factor between the two is the extent of skin involvement with it being <10% in SJS and >30% for TEN.[12]

Cutaneous reactions described in HIV-infected patients focus on the response to individual medications, such as NVP.[13] NVP is a NNRTI and it is a dipyridodiazepinone that binds directly to the viral reverse transcriptase and blocks the RNA polymerase and DNA polymerase activities by causing a disruption of the catalytic site of the enzyme.[14] It is highly specific for HIV-1 reverse transcriptase and does not interfere with human DNA polymerases. NVP has an excellent bioavailability (90%) and a long half-life (25-30 h).[15] Thus, before initiating any other medication NVP should be discontinued for a period of at least three days in order to prevent development of resistance.[1] It is metabolized by the cytochrome p450 system. NVP induces its own metabolism (auto induction) as well as the metabolism of other drugs.[16]

Warren **et al**., reported 20 cases of NVP-associated drug eruptions requiring hospital admission, of which three were fatal.[17] Cutaneous hypersensitivity-associated rash with NVP occurs in the first four weeks of therapy.

In a study by Anton Pon of 263 patients, 166 followed standard recommendations and 94 were started with lower lead in dose. Skin rash associated with NVP was reported in about 8.5% who followed standard recommendations which led to NVP discontinuation while only 2.1% of those who were started with a lower lead in dose reported of rash.[18] These rashes are often accompanied by fever, which usually begins within two to four weeks after starting the treatment and resolves after withdrawing the drug. In all our four cases reported here cutaneous drug hypersensitivity reactions were seen in HIV seropositive patients undergoing treatment with NVP within five to seven days of initiating NVP therapy. It has been shown that a lower lead in dose (200 mg/day instead of the standard 400 mg/day) for the first two weeks of NVP treatment reduces the frequency of cutaneous reaction.[19] Keratinocyte apoptosis has been demonstrated in NVP-associated SJS and it has been postulated that specific cytotoxic lymphocytes probably play a role in the destruction of keratinocyte. This phenomenon could be a part of the immune reconstitution syndrome.[11,1]

The gastrointestinal symptoms associated with NVP may manifest as dysphagia, dyspepsia, and bloody diarrhea. Myocardial injury and hematological complications have also been reported.[3] There are studies that have reported clinical hepatitis as an adverse effect in patients on NVP.[20] Three of our patients presented clinically with elevated liver enzymes and hepatitis. The risk of developing a hepatic hypersensitivity reaction in the first six weeks of NVP therapy is 12-fold more in women with CD4 counts >250/microliter than in those with CD4 counts <250/microliter and fivefold more in men with CD4 counts >400/microliter than in those with CD4 counts <400.[21] Individuals with human leukocyte antigen Bw44, HLA-B12, and HLA-DQB1*0601 appear to be more susceptible to SJS.[22]

Ocular involvement was seen in two of our patients with involvement of the conjunctiva within five to seven days of initiation of the therapy. Similar findings were also reported in the case report by Metry *et al*.[13] Two of our patients were also on ATT and other routine vitamin supplements but their role as a cause of SJS was excluded, since they have been on these medications for a longer duration (3-24 months).

Pollard *et al*., analyzed the safety profile of NVP. Their study involved a total of 906 adults and 468 pediatric patients treated with NVP. Drug-related adverse events were similar in adults and children. The common side-effect observed was mucocutaneous rash and this was seen in both adults and children, but nausea was more common in adults and the occurrence of granulocytopenia was more common in children.[19] Of the four cases reported here one patient was a four-year-old boy, he presented with mucocutaneous rash and hepatitis but did not have granulocytopenia.

As a first line of management HAART was stopped following the development of SJS in all the four patients. These patients were systemically managed with corticosteroids. Oral lesions were managed by topical lignocaine gel. In some patients chlorhexidine mouthwash was added to maintain the oral hygiene. Crusting skin lesions were managed by external paraffin applications. These were in addition to the symptomatic treatment for their presenting complaint and ATT and multivitamin supplements. It is now known that including prednisolone (40-50 mg) daily during the 14-day induction period of ART could be effective in reducing the rate of cutaneous reaction as stated by Kaspar R in his study of 155 patients.[23]

World Health Organization (WHO) guidelines recommend initiation of ART with efavirenz, the only alternative NNRTI available that has similar efficacy to NVP, but a different toxicity profile. Thus despite the recognized toxicities, NVP is one of the most commonly prescribed antiretroviral. The probable reason could be the fact that the generic formulation, including fixed dose combinations, are now available in India and other developing countries at an affordable cost.

In conclusion, we would like to state that patients started with NVP have a potential risk of developing SJS. The oral erythema and ulcerations are usually the initial presenting complaint for which the patient seeks treatment. There are documented reports in the literature where an early diagnosis of SJS could be made due to the presence of oral lesions. Symptomatic management of the oral lesions is necessary in order to enable the patient to have oral feeds to maintain nutritional balance. Increased clinical vigilance is required to identify hypersensitivity reactions like rash and/or other clinical symptoms such as fever, nausea, and abdominal pain, which could occur in the initial six weeks of NVP therapy. Early diagnosis helps the clinician to elude secondary infection and subsequent complications. Laboratory monitoring of liver function tests with elevated hepatic enzymes is mandatory. Once a diagnosis of nevirapine hypersensitivity reaction is made, NVP should be discontinued immediately and not rechallenged.
